# Supplementary material for: Multiplex profiling of 16 immune checkpoints identifies novel serum biomarker panels for breast cancer detection and TNBC stratification: A case-control study
Source: PLoS One. 2026 May 19;21(5):e0348953. doi: 10.1371/journal.pone.0348953 (PMC13186330; doi:10.1371/journal.pone.0348953)
Supplement: S1 Table — (PDF) [file pone.0348953.s001.pdf]

SUPPLEMENTARY TABLE S1

Complete Serum Immune Checkpoint Protein Levels in Breast Cancer Patients and Healthy Controls

IC Protein	Controls: Median (IQR) pg/mL	BC Patients: Median (IQR) pg/mL	p-value [1]	Adjusted p-value [2]	Cohen's d (95% CI) [3]	Direction [4]
**LAG-3**	93,688 (61,450-126,200)	6,008 (3,200-10,500)	<0.001	<0.001	0.89 (0.57-1.21)	↓
**BTLA**	77.2 (45.3-112.0)	40.2 (22.1-65.8)	0.002	0.002	0.67 (0.36-0.98)	↓
**CD80**	14.9 (8.2-22.5)	8.7 (5.1-14.2)	0.003	0.003	0.65 (0.34-0.96)	↓
**GITRL**	58.1 (35.2-85.6)	18.5 (10.2-32.8)	0.003	0.003	0.63 (0.32-0.94)	↓
**GITR**	36.2 (22.5-54.8)	58.4 (38.2-78.5)	0.01	0.16	0.52 (0.21-0.83)	↑
**TLR-2**	472.4 (285.0-688.5)	365.2 (245.0-515.8)	0.01	0.16	0.48 (0.17-0.79)	↓
**CTLA-4**	9.5 (5.8-14.2)	8.1 (4.9-11.8)	0.04	0.64	0.42 (0.11-0.73)	↓
**CD27**	2,110 (1,250-4,850)	2,279 (1,180-4,200)	0.98	1.00	0.02 (-0.28-0.32)	—
**CD28**	612 (385-1,150)	637 (398-1,100)	0.99	1.00	0.01 (-0.29-0.31)	—
**CD40**	667 (485-920)	667 (450-985)	0.20	1.00	0.15 (-0.15-0.45)	—
**CD86**	308 (220-425)	305 (215-410)	0.94	1.00	0.03 (-0.27-0.33)	—
**HVEM**	3,685 (2,450-4,850)	3,586 (2,200-5,100)	0.76	1.00	0.06 (-0.24-0.36)	—
**ICOS**	81.4 (48.5-125.0)	58.4 (35.2-98.5)	0.18	1.00	0.17 (-0.13-0.47)	—
**PD-1**	258 (185-385)	272 (195-398)	0.86	1.00	0.04 (-0.26-0.34)	—
**PD-L1**	18.5 (12.8-26.5)	16.5 (11.2-24.8)	0.18	1.00	0.16 (-0.14-0.46)	—
**TIM-3**	3,194 (2,150-4,850)	3,240 (2,100-4,650)	0.78	1.00	0.05 (-0.25-0.35)	—

BTLA: Band T lymphocyte attenuator; **CD**: Cluster of differentiation; **CTLA-4**: cytotoxic T-lymphocyte–associated antigen 4; **GITR**: glucocorticoid-induced; **GITRL**: glucocorticoid-induced ligand; **HVEM**: herpes virus entry mediator; **ICOS**: Inducible costimulator; **LAG-3**: Lymphocyte-Activation Gene 3; **PD-1**: programmed death-1; **PD-L1**: programmed death-ligand 1; **TIM-3**: T cell immunoglobulin mucin-3; **TLR-2**:Toll like receptor 2

1. p-values calculated using Mann-Whitney U test.
2. Adjusted p-value after Bonferroni correction for 16 comparisons (α=0.003).
3. Effect sizes calculated as Cohen's d with 95% confidence intervals.
4. ↓ indicates downregulation in BC patients; ↑ indicates upregulation in BC patients
