# Supplementary material for: Multiplex profiling of 16 immune checkpoints identifies novel serum biomarker panels for breast cancer detection and TNBC stratification: A case-control study
Source: PLoS One. 2026 May 19;21(5):e0348953. doi: 10.1371/journal.pone.0348953 (PMC13186330; doi:10.1371/journal.pone.0348953)
Supplement: S2 Table — (PDF) [file pone.0348953.s002.pdf]

Supplementary Table S[illegible]

Complete Comparative Analysis of Serum Immune Checkpoint Levels Across Clinic[illegible]

Subgroup	BTLA	CD27	CD28	CD40	CD80/B7-1	CD86/B7-2	CTLA-4	GITR
**AGE**								
≤53 years (n=44)	97.7±174.4	3474±4505	816±661	825±503	12.7±11.7	346±180	10.0±7.9	63.1±37.9
>53 years (n=44)	65.7±77.3	3728±3440	1106±2165	991±677	14.9±24.2	335±231	11.1±9.8	61.4±31.0
p-value	*0.52*	*0.14*	*0.74*	*0.21*	*0.80*	*0.47*	*0.47*	*0.96*
**BMI**								
<25 kg/m² (n=28)	43.6±48.0	4047±3909	976±734	770±409	14.8±12.6	359±200	12.9±9.2	77.6±26.1
≥25 kg/m² (n=60)	87.9±156	3524±4319	749±584	944±691	14.1±21.6	351±217	10.5±9.6*	62.4±36.3*
p-value	*0.27*	*0.31*	*0.11*	*0.58*	*0.11*	*0.72*	***0.03****	***0.02****
**TNBC STATUS**								
Non-TNBC (n=65)	81.6±146	2693±2290	752±558	846±541	11.2±9.3	326±205	9.3±5.5	61.6±29.1
TNBC (n=23)	88.4±108	4930±3707*	3002±4735**	1325±916**	35.1±52.1	369±209	22.7±19.8*	79.8±70.5
p-value	*0.89*	***0.04****	***0.01*****	***0.02****	*0.13*	*0.50*	***0.03****	*0.94*
**ER STATUS**								
ER- (n=18)	69.9±74.3	3686±4317	1875±3142	903±459	19.7±36.6	326±162	13.6±14.1	82.9±48.2
ER+ (n=67)	82.8±156	3006±2826	685±352	891±644	11.8±9.7	334±217	9.2±6.9	59.1±28.8
p-value	*0.75*	*0.81*	***0.03****	*0.50*	*0.68*	*0.81*	*0.75*	***0.03****
**PR STATUS**								
PR- (n=20)	65.3±72.8	4120±4577	1696±3055	1111±840	18.9±34.7	364±216	13.6±13.4	82.3±48.2
PR+ (n=63)	86.1±159	3393±3888	729±506*	819±501	11.9±10.0	320±204	9.8±7.2	56.6±26.4*
p-value	*0.87*	*0.67*	***0.04****	*0.18*	*0.89*	*0.44*	*0.17*	***0.02****
**TUMOR STAGE**								

Early (n=34)	88.4±148	3503±3037	1319±2456	799±434	18.0±28.1	339±196	13.1±12.5	68.4±41.4
Late (n=36)	73.9±130	3409±3636	779±530	988±773	8.6±5.5*	338±217	8.7±3.4	63.2±30.0
p-value	*0.62*	*0.89*	*0.29*	*0.25*	***0.04****	*0.98*	*0.08*	*0.54*
**TUMOR GRADE**								
Low grade (n=43)	79.2±151	3080±3470	761±530	800±433	11.4±11.2	332±189	11.9±10.5	64.6±23.9
High grade (n=28)	87.5±124	5134±5168*	975±901	1147±761*	18.4±29.7	356±226	9.7±7.9	62.2±35.4
p-value	*0.78*	***0.03****	*0.35*	***0.04****	*0.25*	*0.63*	*0.38*	*0.76*
**DISTANT METASTASIS**								
M- (n=40)	89.2±166	3487±3243	1058±2239	990±610	11.5±12.2	344±202	11.2±11.1	52.3±28.1
M+ (n=48)	76.7±118	3626±4549	870±676	730±366	12.6±9.9	330±210	10.1±6.6	67.7±35.4*
p-value	*0.68*	*0.86*	*0.64*	*0.09*	*0.66*	*0.78*	*0.60*	***0.02****
**TREATMENT RESPONSE†**								
Responders (n=54)	84.3±142	3445±3789	912±1645	878±598	13.2±18.5	338±208	9.8±8.2	68.5±38.2
Non-responders (n=18)	76.8±122	3823±4156	1085±2145	1045±725	15.8±25.4	328±198	11.5±10.8	48.3±22.1*
p-value	*0.82*	*0.67*	*0.73*	*0.32*	*0.61*	*0.84*	*0.46*	***0.018****

Abbreviations: BTLA, B and T lymphocyte attenuator; CD, cluster of differentiation; CTLA-4, cytotoxic T-lymphocyte-associated antigen 4; GITR, glucocorticoid-induced TNFR-related protein[illegible] metastasis; M+, distant metastasis present; PD-1, programmed death-1; PD-L1, programmed death ligand-1; PR, progesterone receptor; TIM-3, T-cell immunoglobulin mucin-3; TLR-2, Toll-[illegible]

*Statistical Analysis: Data presented as mean ± SD (pg/mL). Comparisons performed using Mann-Whitney U test for dichotomous variables. *p<0.05, **p<0.01. †Treatment response assess[illegible]*

2

al and Demographic Subgroups in Breast Cancer Patients

GITRL	HVEM	ICOS	LAG-3	PD-1	PD-L1	TIM-3	TLR-2
35.9±45.8	3713±1942	98.8±119	9044±9884	251±137	18.9±13.3	3493±1664	449±262
43.3±54.9	4418±3157	100±141	8142±7283	572±2119	21.1±12.5	3877±2293	449±280
0.57	*0.36*	*0.93*	*0.73*	*0.71*	*0.05*	*0.71*	*0.95*
41.5±24.1	3579±1514	56.3±35.0	7784±9223	276±186	23.6±13.9	3231±1000	454±337
28.6±37.5*	4217±3090	117±153	7843±6289	499±1886	18.7±10.8	3874±2192	864±3305
0.02*	*0.67*	*0.25*	*0.88*	*0.77*	*0.20*	*0.56*	*0.78*
34.5±43.9	3952±2790	95.5±134	7894±7785	239±147	20.5±13.3	3546±2056	431±250
74.1±84.5	4974±1403*	154±127	14626±1390*	2185±5014	15.6±6.2	4920±1716*	3729±8733*
0.24	***0.03****	*0.16*	***0.02****	*0.39*	*0.33*	***0.02****	***0.03****
51.3±74.8	4150±1827	128±140	10539±10610	1058±3380	14.5±6.1	3825±1786	450±307
35.4±44.8	4018±2926	95.0±133	8024±8134	251±153	21.5±13.9	3608±2139	444±265
0.98	*0.35*	*0.30*	*0.21*	*0.39*	***0.01*****	*0.48*	*0.83*
41.3±62.1	5323±4189	111±123	9686±6697	1039±3285	15.6±8.6	4235±2437	1695±5568
37.3±47.4	3655±1933	101±139	7148±6961*	244±152	21.5±14.0	3493±1916	443±272
0.87	*0.06*	*0.28*	***0.04****	*0.99*	***0.02****	*0.29*	*0.42*

52.8±62.0	3643±1686	112±138	8138±7455	302±221	19.8±13.9	5367±4461	1184±4272
32.5±43.4	4628±3652	88.4±121	6803±4709	356±239	18.1±9.5	4638±2563	482±332
0.13	*0.21*	*0.44*	*0.52*	*0.31*	*0.54*	*0.41*	*0.32*
23.4±21.7	3409±1708	92.8±126	7300±6145	287±206	17.2±10.1	3129±1435	363±121
58.7±55.3*	4839±3618	117±148	10165±11099	417±293	23.4±11.7*	4101±2230*	1405±4786*
0.01*	*0.08*	*0.46*	*0.26*	*0.07*	***0.02****	***0.04****	***0.03****
36.2±50.3	3484±1645	107±138	9375±10895	332±230	20.2±14.0	3124±1164	456±312
42.8±51.7	4107±2112	93.0±126	8042±6125	403±283	19.6±11.7	4078±2390	440±229
0.61	*0.33*	*0.62*	*0.58*	*0.23*	*0.85*	*0.07*	*0.83*
41.2±52.5	3889±2567	102±129	8234±8156	375±348	18.2±11.5	3324±1890	445±268
35.8±44.2	4456±3024	89.5±142	9012±9245	462±412	24.6±14.8*	4458±2215*	467±295
0.67	*0.42*	*0.70*	*0.69*	*0.35*	***0.035****	***0.021****	*0.75*

; GITRL, GITR ligand; HVEM, herpes virus entry mediator; ICOS, inducible costimulator; LAG-3, lymphocyte activation gene-3; M-, no distant
-like receptor 2; TNBC, triple-negative breast cancer.

ed after 6 cycles of taxane-platinum chemotherapy; n=72 evaluable patients.
